# A Safe and Efficient Navigation Framework for Ground Vehicles on Uneven Terrain Considering Kinematic Constraints and Terrain Traversability

**DOI:** 10.3390/s26051481

**Published:** 2026-02-26

**Authors:** Jingyao Gai, Zhiyang Guo, Huimin Su, Wang Qing, Kangye Wei, Zhiqiang Cai, Mingzhang Pan

**Affiliations:** School of Mechanical Engineering, Guangxi University, Nanning 530004, China; 2311301017@st.gxu.edu.cn (Z.G.); 2411391105@st.gxu.edu.cn (H.S.); 2511390177@st.gxu.edu.cn (W.Q.); 2411391119@st.gxu.edu.cn (K.W.); 2511300004@st.gxu.edu.cn (Z.C.); pmz@gxu.edu.cn (M.P.)

**Keywords:** navigation framework, hierarchical planning, unmanned ground vehicle, kinematic constraints, terrain traversability

## Abstract

Ground vehicles navigating uneven terrain must simultaneously guarantee motion safety and efficiency. Safety requires that the planned waypoints lie in highly traversable terrain, while ensuring vehicle reachability to these waypoints, which must be kinematically feasible. Efficiency demands fewer detours and smoother paths that avoid excessive vehicle acceleration and steering. However, existing path planning research for uneven terrain fails to comprehensively integrate vehicle kinematic constraints, terrain factors, path smoothness, rollover risk, and total path length. To address this problem, this paper proposes a novel navigation framework. It first integrates terrain slope, flatness, elevation variation, and sparsity to generate a 2D global terrain traversability cost map. Subsequently, a three-phase path planning algorithm integrates A*, guided Rapidly-exploring Random Tree (RRT), and our proposed Kinematic and Terrain-Aware Probabilistic Roadmap (KT-PRM) local re-planning algorithm, which jointly considers multiple factors including ground vehicle kinematic constraints, terrain factors, path smoothness, rollover risk, and path length. This three-phase combination delivers safe, smooth, and short global paths over uneven terrain within a relatively short planning time. Finally, Nonlinear Model Predictive Control (NMPC) is employed for path tracking in the framework. Experiments were conducted in both simulated and real-world uneven terrain environments. The results demonstrated that the three-phase path planning algorithm integrated with our proposed KT-PRM algorithm achieves comprehensive performance in generating safer, smoother, and shorter paths. Our proposed navigation framework achieves safer and more efficient navigation compared with existing navigation frameworks.

## 1. Introduction

Uneven terrain is common in unstructured outdoor environments such as woodland, construction sites and mining areas [1,2,3]. The irregular surface of uneven terrain significantly affects the safety and efficiency of ground vehicle autonomous navigation [4,5,6,7]. The safety of navigation requires the planned waypoints to avoid low traversability terrain, such as steep slopes, highly undulated regions, and pits, while ensuring kinematically feasible paths that guarantee vehicle reachability [8,9,10]. These requirements further demand vehicle stability and rollover prevention for safe path tracking. Efficiency demands fewer detours and path smoothness without excessive vehicle acceleration and steering, which would raise motion cost during path tracking. Therefore, developing a global path planning method that comprehensively and effectively integrates multiple factors, namely vehicle kinematic constraints, terrain traversability, path smoothness, rollover risk, and path length, remains an open challenge.

A key limitation of existing uneven terrain path planning methods is their partial consideration of these factors. Some studies incorporate terrain factors such as slope, flatness, or elevation variation into sampling-based or graph-based planning algorithms (e.g., Rapidly-exploring Random Tree (RRT), A*, Dijkstra) to avoid hazardous regions [11,12,13,14]. Although these terrain-aware algorithms effectively enhance terrain-related safety of global paths, they often ignore strict kinematic constraints. Consequently, the generated paths either incur significant vehicle motion cost when tracked directly via the controller or require heavy smoothing before execution. Moreover, these algorithms neglect the vehicle’s rollover risk during traversal, which corresponds to the vehicle’s motion direction into the local terrain [15,16], thereby compromising the absolute safety of vehicle execution.

Other research formulates planning problems on uneven terrain as nonlinear optimization problems [17,18]. Solving those problems requires complex models, and the solutions are dependent on initial solution quality. Reinforcement learning-based terrain navigation methods [19,20,21,22] have recently emerged, but they rely on extensive training data and high-quality perception, limiting their applicability in real-world outdoor navigation [23].

Overall, despite recent advances in uneven terrain navigation, jointly accounting for vehicle kinematic constraints, terrain traversability, path smoothness, rollover risk, and path length within a computationally efficient navigation framework remains an open challenge.

To address this challenge, we propose a safe and efficient navigation framework. In this framework, a global terrain traversability cost map is first constructed, which fuses slope, flatness, elevation variation, and point cloud sparsity into a unified terrain traversability index. On this map, a globally kinematically feasible and safe path is then generated by a three-phase path planning algorithm, which comprises A*, guided RRT, and a novel local re-planning algorithm, termed Kinematic and Terrain-Aware Probabilistic Roadmap (KT-PRM). Unlike the classic Probabilistic Roadmap (PRM), the proposed KT-PRM considers the vehicle kinematic constraints and integrates terrain traversability cost, motion cost, deviation cost, and rollover cost, accounting for the vehicle’s direction of motion, enabling the planner to produce safe, smooth, and short global paths without a post-smoothing process. The navigation framework is finalized by an NMPC (Nonlinear Model Predictive Control) tracker for real-world deployment.

The main contributions of this paper are summarized as follows:

1. We propose a novel path planning algorithm termed KT-PRM based on the PRM, which comprehensively and effectively incorporates the kinematic constraints of ground vehicles, terrain traversability cost, motion cost, deviation cost, and rollover cost, accounting for the vehicle’s direction of motion. This algorithm introduces an innovative roadmap construction strategy and an extended accumulated cost function in graph search.
2. We propose a novel and comprehensive framework for safe and efficient navigation on uneven terrain. It comprises a global terrain traversability cost map construction algorithm, a three-phase path planning algorithm, and an NMPC controller for path tracking.
3. The proposed navigation framework is validated through both simulated and real-world uneven terrain environments, evaluating its safety, efficiency, and superiority in navigation.

## 2. Related Works

Path planning on uneven terrain demands dealing with complex and highly variable topography while guaranteeing vehicle kinematic feasibility, smooth paths, and high traversal efficiency. Existing research can be grouped into three categories: (1) terrain-aware path planning on uneven terrain, (2) kinematic and dynamic constrained path planning on uneven terrain, and (3) hierarchical planning and PRM variants on uneven terrain. This section reviews the progress and limitations of each category.

### 2.1. Terrain-Aware Path Planning on Uneven Terrain

Incorporating terrain characteristics such as slope, flatness, and point cloud sparsity into path planning algorithms is a common strategy to avoid low traversability regions. Plane-fitting and local geometry features of uneven terrain have been widely used to calculate terrain traversability for sampling-based planners. For example, Jian et al. [11] applied plane-fitting to estimate local terrain slope and integrated terrain traversability into the PF-RRT* algorithm. Probabilistic sampling biased by terrain structure has been introduced in uneven terrain environments such as Gaussian-adaptive samplers [24] and history-aware terrain labeling for navigating unknown terrain [25]. For graph-based planners, some research constructed supervoxel graphs to calculate minimal cost with the Dijkstra graph search algorithms [26] or formulated Traversal Risk Graphs for path optimization in unstructured environments [15]. But vehicle kinematic constraints, path smoothness, and rollover risk were not taken into account for planning in the aforementioned algorithms, affecting tracking effectiveness and safety. Liu et al. [27] combined multi-layer elevation information with a traversability cost module in Hybrid A* planning. A cost function combining terrain metrics and estimated vehicle effort was used in A* or graph-based planners in [28]. These algorithms generated terrain-aware and kinematically feasible paths, but they still neglected rollover risk, and their cost functions in graph search omitted kinematic parameters such as vehicle acceleration and path curvature.

These methods effectively guide planned paths away from low traversability regions. However, they focus solely on terrain factors and basic kinematic feasibility, neglecting comprehensive incorporation of kinematic constraints, path smoothness, rollover risk, and path length. Consequently, some resulting paths violate vehicle physical limits, exhibit low waypoint reachability, and suffer from reduced safety and efficiency during traversal [29], while others are jagged, raising motion cost in path tracking.

### 2.2. Kinematics and Dynamics Constrained Path Planning on Uneven Terrain

Some research emphasizes the compliance with kinematics, dynamics, and curvature constraints of vehicles, which can generate paths that are inherently feasible for Ackermann-steering vehicles or car-like mobile robots. In these algorithms, path generation is formulated as a constrained optimization problem. Savkin et al. [18] minimized fuel consumption on rugged terrain through nonlinear optimization. Xu et al. [30] incorporated curvature and dynamic feasibility constraints into a nonlinear optimization framework for 3D terrain. Wang et al. [31] extended 2D trajectory generation into 3D environments with smoothness, collision, and terrain-related constraints.

Although these optimization-based planners can generate kinematically and dynamically feasible paths, their effectiveness remains constrained by initial solution quality, limiting potential for breakthrough optimization improvements [32,33]. Additionally, it is difficult to construct accurate dynamics models of the robot in contact with the terrain in more complex scenarios [30].

### 2.3. Hierarchical Planning and PRM Variants on Uneven Terrain

Hierarchical or multi-phase navigation frameworks decompose complex path planning tasks into manageable subproblems, including global planning and local refinement. Multi-scale navigation systems have been proposed for semi-structured or partially known environments [34]. Two-stage planners for reconfigurable tracked robots combine global search with detailed local optimization [35]. Shen et al. [36] introduced a three-phase algorithm including A*, RRT, and local trajectory refining to generate trajectories satisfying nonholonomic constraints on 3D terrain. PRM has also been extended to uneven terrain due to its flexibility in incorporating terrain factors during roadmap construction and graph search. Variants of PRM have been developed for long-distance uneven terrain navigation [37], graph search using quantified soil mechanics [38], personalized driving style path planning [39], and semantic belief path planning [40].

Despite these advances, PRM-based algorithms share a critical limitation in path planning on uneven terrain: the sampling process of roadmap construction strategies is point-based, and the edges are typically validated only via collision checking without kinematic constraints. These limitations result in ignoring the vehicle’s actual motion limits, resulting in insufficient feasibility of the planned paths.

In summary, the reviewed algorithms have made significant progress in improving terrain safety and enforcing kinematic feasibility. However, no existing algorithms or navigation frameworks jointly incorporate comprehensive factors, including terrain traversability, path smoothness, path length, and rollover avoidance. This motivates the navigation framework proposed in this paper. By combining the construction of a global terrain traversability cost map, a three-phase path planning algorithm including A*, guided RRT, and a novel local re-planning algorithm termed KT-PRM, which integrates comprehensive factors, our navigation framework enables safe and efficient navigation via a final NMPC tracker on uneven terrain.

The rest of this article is structured as follows. Section 3.1 and Section 3.2 describe the construction of the global terrain traversability cost map and the vehicle kinematic model, including the kinematic constraint model for Ackermann-steering vehicles and a neural network for estimating motion cost during path planning. Section 3.3 and Section 3.4 introduce a three-phase path planning algorithm, in which Section 3.3 details A* and guided RRT as the first two phases in path planning. Section 3.4 details the proposed KT-PRM as the third phase in path planning. Experimental results and discussions are presented in Section 4, encompassing both simulated and real-world environments. This paper ends with conclusions and references.

## 3. Methods

**Figure 1:**
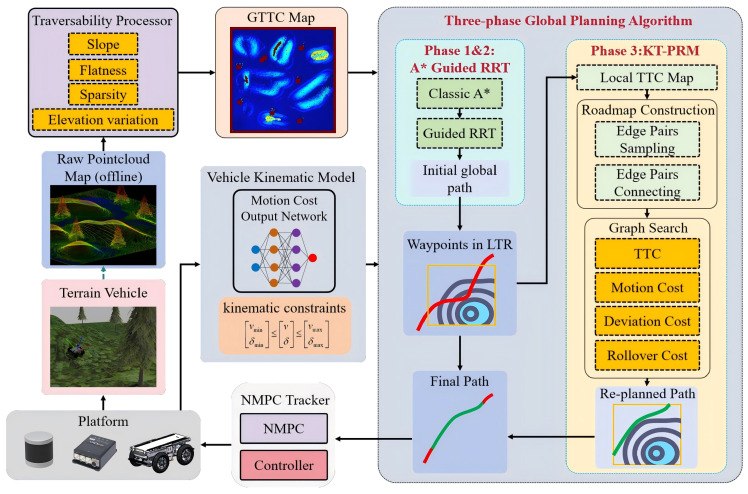
Architecture of the proposed framework. A raw global point cloud map is generated via offline processing of LiDAR data and SLAM, followed by GTTC map processing. A three-phase algorithm generates the final global path, which is tracked on uneven terrain via an NMPC tracker.

Figure 1 illustrates the proposed navigation framework. An initial point cloud map is constructed offline from LiDAR point cloud data via Simultaneous Localization and Mapping (SLAM). This initial map undergoes segmentation algorithms and terrain traversability cost (TTC) calculation through the traversability processor to generate a global terrain traversability cost (GTTC) map. Subsequently, a three-phase path planning algorithm generates global paths, which are tracked using NMPC. Specifically, the three-phase path planning algorithm comprises the following: (1) the classic A* generates a global obstacle avoidance path, (2) the A*-guided RRT generates an initial global path that reaches the goal while considering vehicle kinematic constraints, and (3) a KT-PRM algorithm re-plans the waypoints of the initial global path that lie within low traversability regions (LTRs). The re-planned waypoints and other waypoints together form the final global path.

### 3.1. GTTC Map Construction

**Figure 2:**
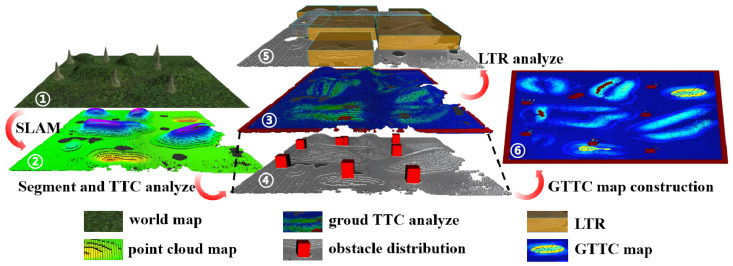
Map processing workflow. Taking the simulation environment map (①) as an example, a global point cloud map (②) is obtained via SLAM, followed by segmentation into ground point cloud and obstacle point cloud. The ground point cloud undergoes ground traversability cost analysis on a grid map, with results visualized on an octree map (③). Concurrently, obstacle distribution (④) and LTR distribution (⑤) are derived. Finally, the ground traversability cost and obstacle distribution are fused to generate the GTTC map (⑥).

The map processing workflow is shown in Figure 2. The initial point cloud map constructed via SLAM is segmented into ground point cloud and obstacle point cloud using a region growing segmentation algorithm. A 2D grid map is generated from the ground point cloud, with each grid storing the TTC value corresponding to its center point to form a GTTC map. Obstacles are approximated as rectangles to obtain the corresponding 2D grid location. The resolution of the GTTC map in this paper is 0.2 m.

For each 3D coordinate point $x_{i}$ in the ground point cloud, a square kernel $K(x_{i})$ is defined as the set of points within a square region centered at $x_{i}$ with side length $L_{d}$. Using the SVD algorithm, a plane is fitted in $K(x_{i})$, and the corresponding unit normal vector is represented as $n_{i}=\left(n_{i}^{x},n_{i}^{y},n_{i}^{z}\right)$. This paper employs four metrics, including slope (*s*), flatness (*f*), elevation variation (*g*), and sparsity (λ), to evaluate the terrain traversability. These four metrics are defined as follows:

(1) $$\begin{array}{c} s=\arccos(n_{i}^{T}\;\cdot \;k), \end{array}$$

(2) $$\begin{array}{c} f=\frac{1}{N}\sum_{j=1}^{N}e^{|n_{i}^{T}\;\cdot \;x^{j}|}, \end{array}$$

(3) $$\begin{array}{c} g=z_{\max}-z_{\min}, \end{array}$$

(4) $$\begin{array}{c} \lambda =\left\{\begin{array}{cc} 1, & r_{i}>r_{\max}\\ \frac{r_{i}-r_{\min}}{r_{\max}-r_{\min}}, & r_{i}\in \left[r_{\min},\;r_{\max}\right]\\ 0, & \mathrm{otherwise} \end{array}\right., \end{array}$$

where *k* is the unit vector in 3D Euclidean space perpendicular to the xy plane. $x^{j}$ is the local vector from the center point to all points in $K(x_{i})$. Flatness measures the degree of terrain undulation, while elevation variation quantifies height variation within a point’s local area. Variables $z_{min}$ and $z_{max}$ represent the minimum and maximum elevations within the coordinate point’s local range, respectively. Sparsity $r_{i}$ denotes the proportion of vacant parts in $K(x_{i})$. $r_{min}$ and $r_{max}$ represent the minimum and maximum acceptable vacant ratio, respectively. The presence of concentrated empty parts indicates the existence of pits on the ground surface.

TTC, denoted as $J_{t}$, is the weighted sum of slope, flatness, elevation variation, and sparsity. A higher TTC indicates poorer traversability at that point. The TTC is calculated as follows:

(5) $$J_{t}=\alpha_{1}\;\cdot \;s+\alpha_{2}\;\cdot \;f+\alpha_{3}\;\cdot \;g+\alpha_{4}\;\cdot \;\lambda ,$$

where $\alpha_{1}$, $\alpha_{2}$, $\alpha_{3}$ and $\alpha_{4}$ represent the corresponding weights for each term. Specifically, different terrain characteristics will lead to different types of vehicle navigation failure risks: high slope (s↑) increases the risk of vehicle rollover, low flatness (f↑) increases the risk of bumpy motion and unstable steering, high elevation variation (g↑) increases the risk of abrupt pitch changes and loss of control, and high sparsity (λ↑) increases the risk of vehicle drop-in and collision. The weight allocation is mainly determined intuitively by observing terrain characteristics in specific environments and analyzing the types of vehicle navigation failures that will be caused and their corresponding failure risk levels, following the main principle that a higher weight is assigned to the terrain characteristic that induces a higher navigation failure risk. Based on this principle, we provide two sets of weight allocations for the simulated environments and real-world environments used in this paper, considering the differences in the distribution of terrain characteristics between these two types of environments. The specific weight parameters are presented in Section 4.1 of the experimental results and discussion.

After obtaining the GTTC map, grid cells with TTC exceeding the set threshold are clustered using Density-Based Spatial Clustering of Applications with Noise (DBSCAN). Each clustered grid area is then evaluated for its two-dimensional range, enclosed within a bounding box. By expanding the bounding box by a certain length in two directions, a 2D distribution of LTRs is obtained.

### 3.2. Vehicle Kinematic Model Construction

#### 3.2.1. Bicycle Model and Vehicle Motion Representation in Global Coordinates

All motions discussed herein are described in a 2D plane. The Ackermann-steering vehicle can be modeled as a rear-axle-referenced bicycle model. Control input consists of rear-wheel speed *v* and front-wheel steering angle δ. State variable comprises the world-coordinate-based global position (*x*, *y*) and the yaw angle θ. The vehicle wheelbase is denoted as *L*. The kinematic model is given by the following:

(6) $$\left[\begin{array}{c} \dot{x}\\ \dot{y}\\ \dot{\theta } \end{array}\right]=v\;\cdot \;\left[\begin{array}{c} \cos(\theta )\\ \sin(\theta )\\ \frac{\tan(\delta )}{L} \end{array}\right].$$

**Figure 3:**
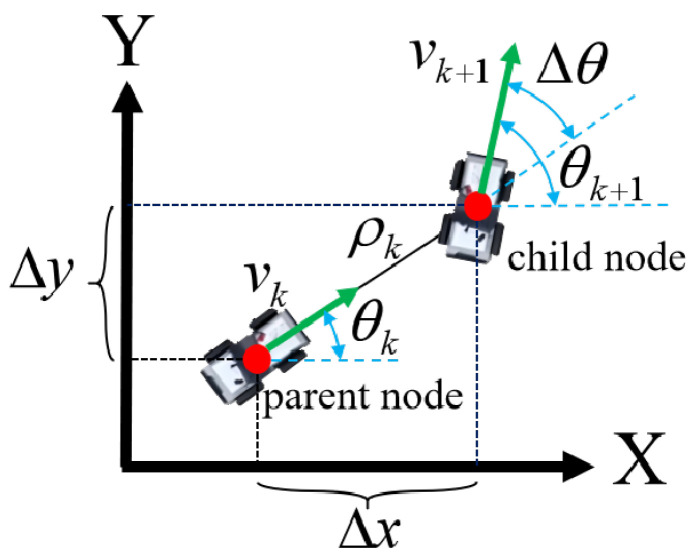
Vehicle kinematic constraints. The global coordinate system is defined as an X-Y coordinate system. The two red nodes represent the parent and child nodes. After moving from the parent node to the child node, the variation in yaw angle is constrained by the distance between the two nodes.

The establishment of the global coordinate system for global path planning is shown in Figure 3. Within the global coordinate system, node sampling is performed by discretizing the bicycle model over time.

#### 3.2.2. Kinematic Constraints and Computation of Motion Cost

Based on the mechanical limits of the vehicle’s actuators, the control input bounds are set as follows:

(7) $$\left[\begin{array}{c} v_{\min}\\ \delta_{\min} \end{array}\right]\leq \left[\begin{array}{c} v\\ \delta \end{array}\right]\leq \left[\begin{array}{c} v_{\max}\\ \delta_{\max} \end{array}\right].$$

From the kinematic model (6), the maximum variation of yaw angle is proportional to velocity and constrained by the maximum front-wheel steering angle. Considering that large front-wheel steering angles under uneven terrain conditions are detrimental to safe vehicle motion, we have imposed restrictions on the variation of the yaw angle and defined a threshold $\Delta \theta_{t}$. The range of the variation of the yaw angle at corresponding velocity *v* is thus defined as follows:

(8) $$\left\{\begin{array}{c} \left|\Delta \theta \right|\leq v\;\cdot \;\frac{\tan(\delta_{\max})}{L}\\ \left|\Delta \theta \right|\leq \Delta \theta_{t} \end{array}\right..$$

In the global coordinate system, after discretizing the vehicle motion time to obtain several states, the path-tracking problem is formulated as a discrete-time optimal control problem (OCP), which can be expressed as follows:

(9) $$\mathrm{minimize}\;\;J=\;{\left\|x-x_{\mathrm{ref}}\right\|}_{Q}^{2}+{\left\|u\right\|}_{R}^{2},$$

where *J* is the cost function, *x* is the state, $x_{\mathrm{ref}}$ is the reference state, *u* is the control input, *Q* is the state weighting matrix, and *R* is the control weighting matrix. The OCP is solved, subject to the kinematic model (6) and kinematic constraints ((7) and (8)).

The optimal control input obtained from solving the OCP is used to calculate the motion cost. Each state corresponds to a node. The motion cost from the parent node to the child node is calculated as follows:

(10) $$J_{mo}=\omega_{\rho }\;\cdot \;\rho^{2}+\omega_{\theta }\;\cdot \;{\left(\Delta \theta \right)}^{2}+\omega_{c}\;\cdot \;\left(v^{2}+\delta^{2}\right),$$

in which the first and the second terms penalize the travel distance and the variation of the yaw angle, respectively. The third term regulates the control effort. The parameters $\omega_{\rho }$, $\omega_{\theta }$, and $\omega_{c}$ are the corresponding weights for each term. In this paper, the weights are set to 0.4, 0.4, and 0.2, respectively. The equal weights of 0.4 ensure balanced penalization of the travel distance and the variation of the yaw angle, while the lower weight of 0.2 on control effort is due to these factors being less critical for path smoothness in off-road uneven terrain.

Since in our developed path planning algorithm, frequent computation of motion cost is required, and solving OCPs via nonlinear programming requires high computational cost, we employ a neural network-based method to directly output motion cost value based on two input parameters, including the vehicle travel distance ρ and the variation of yaw angle Δθ.

The dataset for training the network was created through grid sampling. The range and step size for sampling are shown in Table 1. The ground truth of corresponding motion cost was calculated through solving the OCP in Equation (9) via Interior Point OPTimizer (IPOPT) and applying the optimal control to Equation (10). A dataset of 28,000 samples was created in this study and divided into training, validation, and testing sets at ratios of 70%, 15%, and 15%, respectively. This network featured two hidden fully-connected layers, each containing 100 neurons, with Rectified Linear Unit (ReLU) activation functions between layers.

The average accuracy is used to evaluate the performance of the neural network model, as shown below:

(11) $$\frac{1}{N}\sum_{i=1}^{N}\left(1-\frac{|k_{i}-{\tilde{k}}_{i}|}{k_{i}}\right),$$

where *N* is the total number of samples in the testing dataset, $k_{i}$ is the ground-truth motion cost for the *i*th data, and ${\tilde{k}}_{i}$ is the motion cost output by the neural network. The mean accuracy achieved is 99.46%, satisfying the requirement for precise cost estimation. Furthermore, we compared the runtime of the IPOPT-based method against the network-based method on 30,000 newly constructed cases. The time statistics are summarized in Table 2. The neural network-based method reduced the time per motion cost output by approximately 97.69% compared with the IPOPT-based method.

### 3.3. Initial Global Path Generation

This section covers the first and second phases of the three-phase algorithm, which generate length-suboptimal paths that satisfy vehicle kinematic constraints. This method is primarily divided into two steps. First, an A* algorithm was applied to a binary gridmap representing only obstacle distribution to generate the safe and shortest path from the start to the goal. Then, a guided RRT algorithm was applied to refine the path to satisfy the kinematic constraints. The A*-guided sampling details are as follows (Table 3).

**Figure 4:**
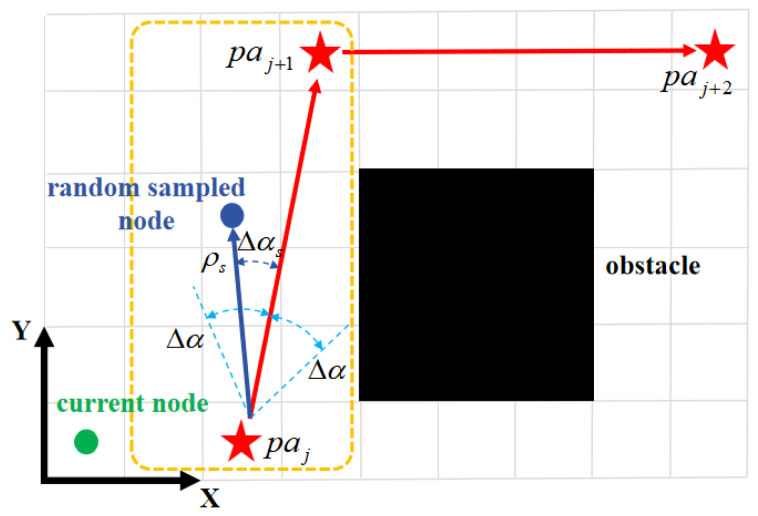
Schematic of A*-guided sampled node generation. The gray grid represents the guided RRT sampling grid, black squares denote grid cells containing obstacles, red pentagrams indicate waypoints planned by A*, and the dark blue dot represents the randomly sampled node based on the value of $\rho_{s}$ and $\Delta \alpha_{s}$. This node will guide the current RRT node (green dot) to sample a new RRT node.

A*-guided sampling proceeds sequentially. The principle is as follows: based on the ratio $k_{p}$ of map resolution between the binary gridmap used in the A* algorithm and the GTTC map, the A*-guided node advances to the next A* waypoint after every $k_{p}$ nodes generated by the guided RRT. The hyperparameters of the sampling process in the A*-guided RRT are configured as shown in Table 3. The A*-guided sampling process is illustrated in Figure 4, with sampled input parameters following these distributions:

(12) $$\begin{array}{c} \rho_{s}∼U\left(\rho_{s,\min},\rho_{s,\max}\right), \end{array}$$

(13) $$\begin{array}{c} \Delta \alpha_{s}∼U\left(\Delta \alpha_{s,\min},\Delta \alpha_{s,\max}\right), \end{array}$$

where $\rho_{s}$ is the sampled distance, and $\Delta \alpha_{s}$ is the sampled variation of the yaw angle. They jointly determine the coordinates of the randomly sampled node on the GTTC map.

Algorithm 1 describes the complete A*-guided RRT algorithm. This algorithm is similar to the RRT proposed by Shen [36], with several modifications made in the functions **FindNearestNode** and **Steer**, and a new function **CheckinLTR** was added to integrate terrain traversability.

**Algorithm 1:** A*-guided RRT

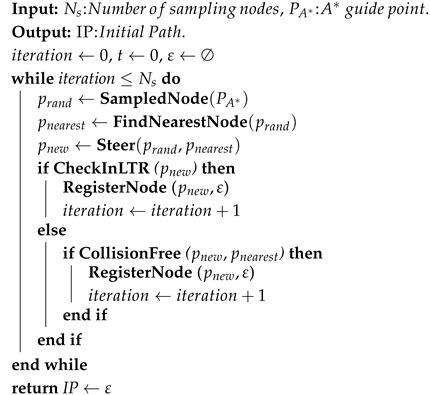



The **FindNearestNode** function only calculates Euclidean distance without involving complex kinematic connecting costs. The **CheckinLTR** function determines whether the node is within the LTRs. The **CalculateCost** function is removed to skip motion cost calculation between nodes. These simplifications were made to enable rapid path search that satisfies reaching the destination while meeting kinematic constraints, without considering obstacle avoidance in LTRs and motion cost comparisons.

The modified **Steer** function (Algorithm 2) is to ensure that the vector from $p_{nearest}$ to $p_{new}$ satisfies kinematic constraints. Additionally, we modified the **Steer** function to account for terrain factors. When sampling in LTRs, we employ a strategy using half the GTTC map resolution as the sampling distance. This ensures sufficiently dense node generation within LTRs to serve as reference waypoints for local path re-planning.

After these two steps, the A* and guided RRT, an initial global path is generated, which is kinematically feasible and of suboptimal length. This initial path maintains dense waypoints within LTRs while avoiding obstacles in regions outside LTRs.

### 3.4. Local Path Re-Planning

This section describes our proposed algorithm for local path re-planning, termed KT-PRM, as the third phase of path planning. Based on the initial global path obtained in A*-guided RRT, KT-PRM can re-plan a local path to generate a safe, smooth, and short path that satisfies kinematic constraints. Given the high calculation cost of re-planning all waypoints of the initial global path and the limited benefits of TTC optimization on relatively flat terrain, local re-planning is restricted to waypoints within LTRs. The KT-PRM algorithm includes roadmap construction and graph search.

#### 3.4.1. Roadmap Construction

As the first step in local path re-planning, a roadmap is constructed as a graph comprising all vertices and edges satisfying the kinematic constraints. The roadmap construction procedure is detailed as follows:

**Algorithm 2:** Modified Steer Function

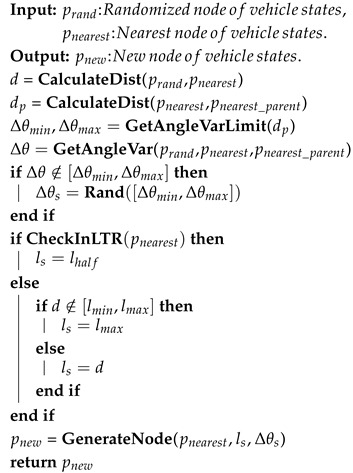



Let the initial path generated by A*-guided RRT be denoted as $P_{init}$, which includes waypoints $p_{0}$, $p_{1}$,…, $p_{n}$. The *k*th LTR in the GTTC map is denoted as $T_{k}$. Therefore, the set of waypoints within the LTR $T_{k}$ is denoted as $P_{k}$, which is defined as follows:

(14) $$P_{k}=\left\{for\;all\;p_{j}\in P_{init}|p_{j}\subset T_{k}\right\},$$

whereas the set of all waypoints in the high-traversability region (HTR) is denoted as $P_{h}$.

**Figure 5:**
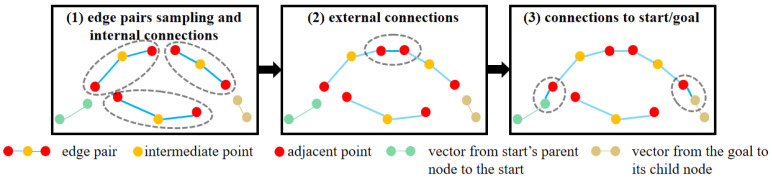
Roadmap construction process. The gray dashed ellipse boxes indicate the ongoing operations of the corresponding step.

The inputs to the roadmap construction algorithm include the waypoints $P_{k}$ and the corresponding local TTC map. The start and goal points in the local TTC map are determined from the waypoints $P_{k}$. The local TTC map is extracted from the GTTC map, enclosing all waypoints in the $P_{k}$. The cost values stored in the local TTC map undergo normalization processing. The roadmap construction process is illustrated in Figure 5 and detailed in Algorithm 3, which mainly consists of three steps: (1) edge pair sampling (**SampleKiEdge** function) and internal connections of edge pairs (**InternalConnect** function); (2) external connections of edge pairs (**ExternalConnect** function); (3) connections to start/goal (**ConnectStart** and **ConnectGoal** function).

**Algorithm 3:** Roadmap Construction

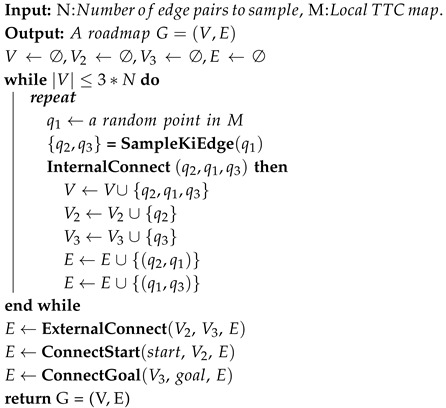



**Figure 6:**
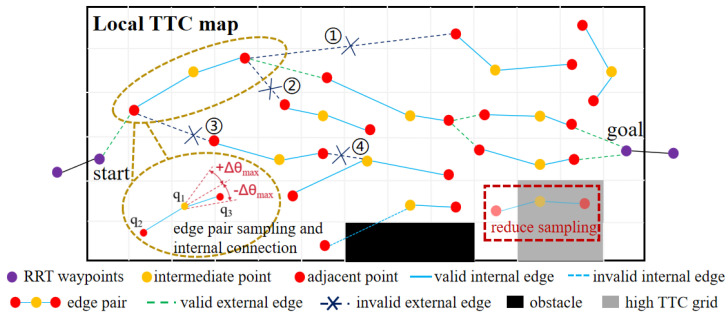
Schematic of edge pairs sampling and connections. The edge pair that traverses the obstacles is rejected. The probability elimination process reduces sampling in regions with higher TTC. External connections of edge pairs shall not result in the following connection outcomes: 1. exceeding the maximum range of travel distance; 2. exceeding the range of the yaw angle variation; 3. connecting vertices within the same vertex set; 4. involving intermediate vertices of the edge pairs.

The first step of roadmap construction is sampling edge pairs and building internal connections, which is depicted in Figure 6. For each point $q_{1}$ sampled on the local TTC map, a function **SampleKiEdge** is employed to generate two adjacent points on either side of $q_{1}$. These two points ($q_{2}$ and $q_{3}$) are quickly ranked according to their Manhattan distance to the start point on the local TTC map, while $q_{1}$, $q_{2}$, and $q_{3}$ satisfy the following constraints:

(15) $$\left\{\begin{array}{c} |∠\left(q_{2},q_{1},q_{3}\right)-\pi |\leq \Delta \theta_{max}\\ \left|\right|q_{2}-q_{1}{\left|\right|}_{2}\leq \rho_{max}\\ \left|\right|q_{3}-q_{1}{\left|\right|}_{2}\leq \rho_{max} \end{array}\right.,$$

where $\Delta \theta_{max}$ is calculated using Equation (8), and $\rho_{max}$ is related to the vehicle’s maximum speed. These guarantee the satisfaction of the kinematic constraints.

An edge is formed by connecting two points. Edges $\left(q_{2},q_{1}\right)$ and $\left(q_{1},q_{3}\right)$ form an edge pair. The collision with the obstacles is then checked by verifying whether either edge of this edge pair intersects with the obstacle regions on the local TTC map. In addition, to ensure edge pairs are concentrated in areas with higher traversability, a probabilistic elimination process is applied based on the TTC value of each edge pair. The probability for elimination is as follows:

(16) $$k\left(r\right)=\left\{\begin{array}{cc} \lambda_{1}, & \mathrm{if}\;\;\sum_{i=1}^{3}J_{t}\left(q_{i}\right)\geq 3\;\cdot \;J_{t}^{\mathrm{th}}\;\;\mathrm{or}\;\;J_{t}\left(q_{i}\right)\geq J_{t}^{\mathrm{th}},\;i=1,2,3\\ \lambda_{2}, & \mathrm{otherwise} \end{array}\right.,$$

where $J_{t}$ is the TTC value (Equation (10)) of the corresponding point after normalization on the local TTC map, and $J_{t}^{\mathrm{th}}$ is the threshold for normalized local TTC value, $J_{t}^{\mathrm{th}}\in \left[0,1\right]$. $\lambda_{1}\in \left[0,1\right]$, $\lambda_{2}\in \left[0,1\right]$. Set $\lambda_{1}>\lambda_{2}$ to form edge pair sampling favor areas with lower TTC. In our study, we set $\lambda_{1}=0.8$, $\lambda_{2}=0.2$, and $J_{t}^{\mathrm{th}}=0.7$. If an edge pair is rejected due to the elimination process or collision checking, resampling is performed until *N* edge pairs are obtained.

When an edge pair is retained, its three points are added to the vertex set *V*, which already contains the start and goal points, and its two edges are added to the edge set *E*. The points $q_{2}$ and $q_{3}$ of the edge pair are added to the vertex set $V_{2}$ and $V_{3}$, respectively, for subsequent external connections.

The second step of roadmap construction is building external connections; this involves connecting the vertices in $V_{2}$ to the vertices in $V_{3}$. Kinematic feasibility and collision are checked before vertice connection. External connection scenarios are also shown in Figure 6. When an external connection of edge pairs succeeds, the external connecting edge is added to the edge set *E*.

The third step of roadmap construction is building connections between all edge pairs with the start and the goal; this step is similar to building external connections. The kinematic feasibility of the new connections is verified using vectors from the start’s parent node to the start and from the goal to its child node.

After these three steps, a roadmap G=(V,E) is constructed, encompassing all vertices and kinematically feasible edges.

#### 3.4.2. Graph Search

As the second step of local path re-planning, graph search is applied to the constructed roadmap G=(V,E) to obtain the optimal path connecting the start and the goal. While our graph search builds upon the A* algorithm as a heuristic optimal search, existing cost functions are limited to optimizing path length exclusively. We address this limitation by proposing an extended accumulated cost function that integrates terrain factors, path smoothness, rollover risk, and path length into a unified metric. Let $v_{i}\in V$ denote the parent vertex during the graph search process and $v_{i+1}\in V$ denote the candidate vertex for expansion from $v_{i}$. When expanding from $v_{i}$ to $v_{i+1}$, the accumulated cost $C(v_{i+1})$ from the start vertex to $v_{i+1}$ is calculated as follows:

(17) $$C\left(v_{i+1}\right)=C\left(v_{i}\right)+\omega_{t}\;\cdot \;C_{t}+\omega_{m}\;\cdot \;C_{m}+\omega_{r}\;\cdot \;C_{r}+\omega_{d}\;\cdot \;C_{d},$$

where $C_{t}$, $C_{m}$, $C_{r}$, $C_{d}$ are the terrain cost, complete motion cost, rollover cost, and deviation cost from $v_{i}$ to $v_{i+1}$, respectively. The parameters $\omega_{t}$, $\omega_{m}$, $\omega_{r}$, and $\omega_{d}$ are the corresponding weights for each term. In this paper, the weights are set to 0.5, 0.2, 1, and 0.3, respectively. This allocation of weights prioritizes terrain factors, followed by path length and path smoothness. The rollover cost weight is set to 1 because the rollover cost value is either *∞* or 0, ensuring that any non-zero rollover contribution is heavily penalized. Cost terms $C_{t}$, $C_{m}$, $C_{r}$, and $C_{d}$ penalize terrain non-traversability, motion effort, rollover risk, and deviation from the length-suboptimal reference path, respectively, which are detailed as follows:

The terrain cost $C_{t}$ is computed as the sum of the normalized TTC values at $v_{i}$ and $v_{i+1}$:

(18) $$C_{t}=J_{t}\left(v_{i}\right)+J_{t}\left(v_{i+1}\right).$$

The motion cost $C_{m}$ represents the complete cost for path smoothness. $C_{m}$ is computed as follows:

(19) $$C_{m}=\omega_{mo}\;\cdot \;J_{mo}+\omega_{a}\;\cdot \;{\left(d_{2}-d_{1}\right)}^{2}+\omega_{\kappa }\;\cdot \;\frac{2\sin\theta }{d_{1}+d_{2}},$$

where $J_{mo}$ is generated by a neural network, which penalizes travel distance and heading variation and regulates control effort (speed and front-wheel steering angle). The second and third terms penalize vehicle acceleration and approximate curvature of the paths, respectively. Specifically, $d_{1}$ and $d_{2}$ denote the lengths of two adjacent path segments, with θ representing the angle between them. The parameters $\omega_{mo}$, $\omega_{a}$, and $\omega_{\kappa }$ are the corresponding weights for each term. In this paper, the weights are set to 0.5, 0.15, and 0.6, respectively, achieving a well-balanced contribution of each cost term within the motion cost.

**Figure 7:**
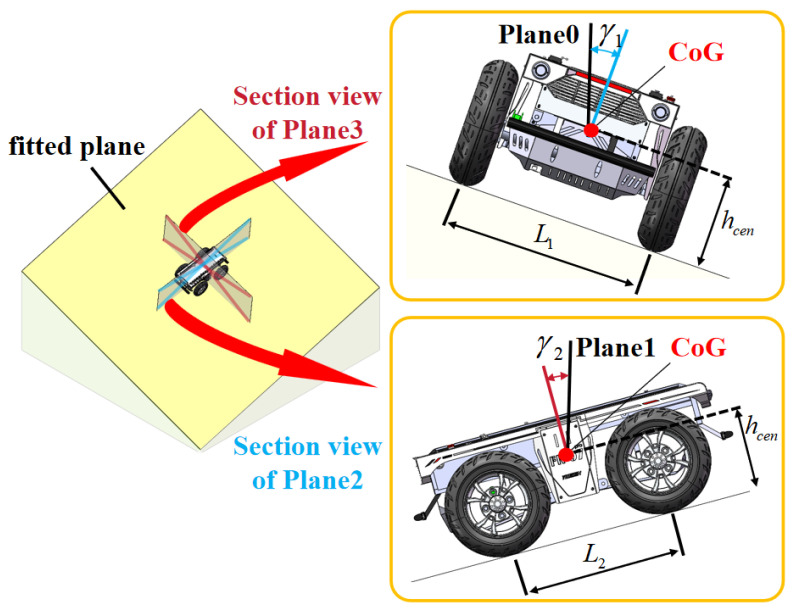
Vehicle’s posture model on the fitted plane. Key parameters are listed below. $h_{cen}$: distance from CoG to the fitted plane. $\gamma_{1}$: angle between Plane2 and Plane0. $\gamma_{2}$: angle between Plane3 and Plane1. $L_{1}$: track width. $L_{2}$: wheelbase. The reference planes are defined as follows: Plane0 passes through the velocity vector and is perpendicular to the theoretical horizontal plane. Plane1 is orthogonal to Plane0 and passes through the CoG. Plane2 passes through the velocity vector and the normal vector at the CoG. Plane3 is orthogonal to Plane2 through the CoG.

The rollover cost $C_{r}$ is evaluated based on the vehicle’s wheelbase, track width, center-of-gravity (CoG) height, and posture at the corresponding vertex. The vehicle’s posture is derived from the locally fitted plane, assuming simultaneous contact of both front and rear wheels with the plane. The vehicle’s posture model on the fitted plane is illustrated in Figure 7.

According to the vehicle’s posture model in Figure 7, $C_{r}$ is set as follows:

(20) $$C_{r}=\left\{\begin{array}{cc} \infty , & \mathrm{if}\;\gamma_{1}\geq \arctan\;\left(\frac{L_{1}}{2\;\cdot \;h_{\mathrm{cen}}}\right)\;\mathrm{or}\;\gamma_{2}\geq \arctan\;\left(\frac{L_{2}}{2\;\cdot \;h_{\mathrm{cen}}}\right)\\ 0, & \mathrm{otherwise} \end{array}\right..$$

For the FR-07 Pro Ackermann-steering vehicle, which was used for the experiments in this paper ($L_{1}=0.645$ m, $L_{2}=0.66$ m, $h_{\mathrm{cen}}=0.57$ m), this gives the static rollover angle threshold $\gamma_{max}\approx 30^{\circ }$. Note that this paper considers only the geometric static rollover threshold, but off-road conditions introduce terrain and tyre deformation as well as transient lateral accelerations, both of which can reduce the actual rollover limit below the static value $\gamma_{max}$ [41,42,43]. Terrain and tyre deformation, as well as transient lateral accelerations, are not explicitly modeled in this paper. To account for this, we adopted 24° as the maximum allowable roll and pitch angle, derived from the static rollover threshold plus a 6° safety margin [44]. A full and accurate dynamic rollover model will be developed in future work.

The deviation cost $C_{d}$ quantifies how far $v_{i}$ and $v_{i+1}$ deviate from the reference waypoints $P_{k}$, which is calculated as follows:

(21) $$C_{d}=\frac{1}{L_{diagonal}}(\sum_{j=1}^{n}{\left\|v_{i}-p_{kj}\right\|}_{2}+\sum_{j=1}^{n}\|v_{i+1}-p_{kj}{\|}_{2}),$$

where $\|v_{i}-p_{kj}{\|}_{2}$ denotes the Euclidean distance from the vertex $v_{i}$ to the *j*th point of reference waypoints $P_{k}$ on the local TTC map. *n* is the number of waypoints in $P_{k}$. $L_{diagonal}$ is the diagonal length of the local TTC map.

The evaluation function $J(v_{i+1})$, integrating the accumulated cost and heuristic estimate, guides vertex expansion during graph search and is calculated as follows:

(22) $$J\left(v_{i+1}\right)=C\left(v_{i+1}\right)+{\left\|v_{i+1}-v_{goal}\right\|}_{2},$$

where $C(v_{i+1})$ denotes the accumulated cost, and $v_{goal}$ is the goal vertex on the roadmap. The second term corresponds to the heuristic function, which ensures that the evaluation function incorporates the minimum distance to the goal.

Through the graph search on the roadmap, the re-planned path $P_{re}$ from the start vertex to the goal vertex is obtained. Leveraging our extended accumulated cost function, $P_{re}$ has the minimized total cost, which encompasses TTC, complete motion cost, rollover cost, and deviation cost, thereby yielding a kinematically feasible, safe, smooth, and short path that avoids low-traversability regions. The final global path is obtained by combining all re-planned paths $P_{re}$ with the paths $P_{h}$ located in HTR, which are not re-planned.

## 4. Experimental Results and Discussion

### 4.1. Experimental Setup

Experiments were conducted to evaluate the performance of the proposed navigation framework in global path planning and tracking on uneven terrain within both simulated environments and real-world environments. Specifically, in simulated environments, our algorithm for global path planning was compared with the state-of-the-art algorithms, and the effectiveness of the proposed KT-PRM in the three-phase algorithm was demonstrated. In real-world experiments, the proposed navigation framework was compared with other frameworks to verify its safety and efficiency.

#### 4.1.1. Simulated Environments

**Figure 8:**
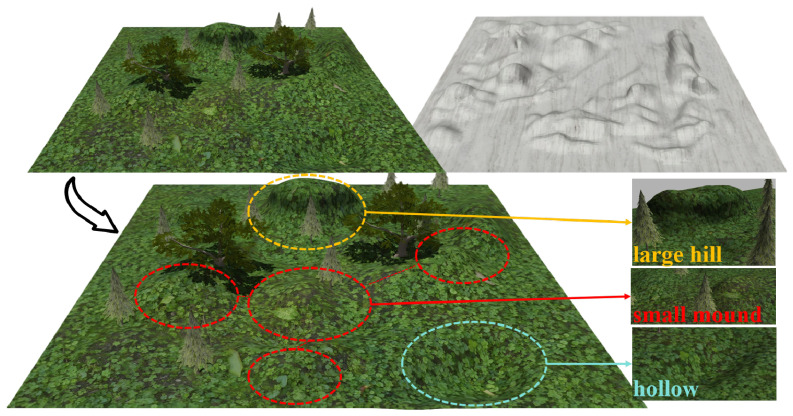
Two simulated environments. (**Top left**): woodland environment; (**top right**): mining site environment. (**Bottom**) panel illustrates woodland terrain details, including large hills, small mounds, and hollows.

Two types of simulated environments were constructed in Gazebo: woodland environments and mining site environments. The woodland environment features typical uneven terrain characteristics, with detailed topography shown in Figure 8. The mining site environment similarly contains areas with slopes and high unevenness, but with a higher density distribution. For each type of environment, we prepared five maps with a size of 40 m × 40 m, each with distinct terrain distribution characteristics, yielding a total of ten maps across both environments.

Our algorithm was compared against PUTN [11] and T-Hybrid A* [27] on the aforementioned maps using three metrics for the path planning and three for simulated vehicle execution. The metrics for path planning were angle-over-length (AOL), total path length, and the average TTC of waypoints. The AOL, defined in [45], quantifies path smoothness as the ratio of total heading change to path length. The TTC of a waypoint was calculated for all algorithms using identical slope, flatness, and sparsity formulas with the same weight distribution. The metrics for execution were navigation success rate (defined as the proportion of the vehicle’s uninterrupted executions from start to goal out of total trials), average roll angle, and average pitch angle (sampled at 0.5 s intervals). The simulation experiment parameters are summarized in Table 4. The simulation experiments were executed on an Ubuntu 20.04 system equipped with an i7-13650HX processor and 24 GB RAM.

#### 4.1.2. Real-World Environments

Three testing environments were used for evaluating our navigation framework: a 30 m × 30 m woodland area with concentrated mounds, rocks, and trunk obstacles; a 50 m × 50 m uneven grassland with small slopes and dense undulations; and a 50 m × 20 m arch bridge setting spanning an impassable pond. The vehicle was required to avoid obstacles while considering terrain factors in the woodland, assess terrain traversability for efficient path planning in the grassland, and execute safe crossing maneuvers between flat regions in the bridge environment.

**Figure 9:**
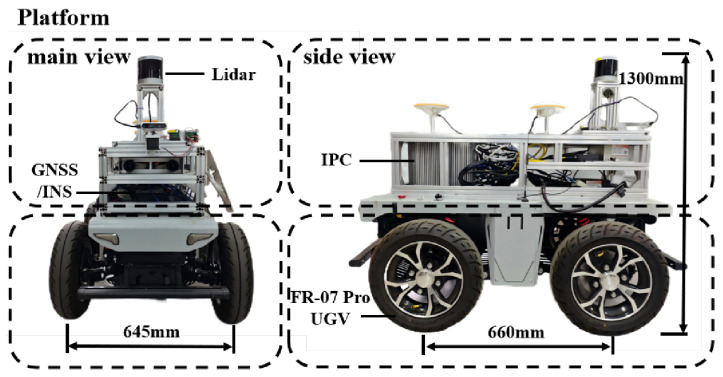
The vehicle platform for real-world experiments. It is equipped with a LiDAR, an IPC, and the GNSS/INS.

In the real-world experiments, we used a ground vehicle with Ackermann steering (FR-07 Pro, YUHESEN, China) as a testing platform (Figure 9). It featured a LiDAR sensor (Helios 32, RoboSense, China) for 3D terrain mapping. The positioning was obtained using a GNSS/INS module. The proposed navigation framework was deployed on the DTB-3079-C246 vehicle-mounted industrial personal computer (IPC) with an Intel Core i7-9700 processor. The real-world experiment parameters are summarized in Table 5.

The 3D maps for path planning were constructed using the A-LOAM [46] algorithm in both simulated and real-world environments.

### 4.2. Simulation Environment Experiments

#### 4.2.1. Performance Comparison of Path Planning Algorithms on Uneven Terrain

For each simulated map, ten path planning experiments were conducted with the start and goal points strategically positioned at map edges. A total of 100 path planning experiments were conducted among our algorithm, PUTN, and T-Hybrid A*. The distance from start to goal was constrained to 35–45 m.

**Figure 10:**
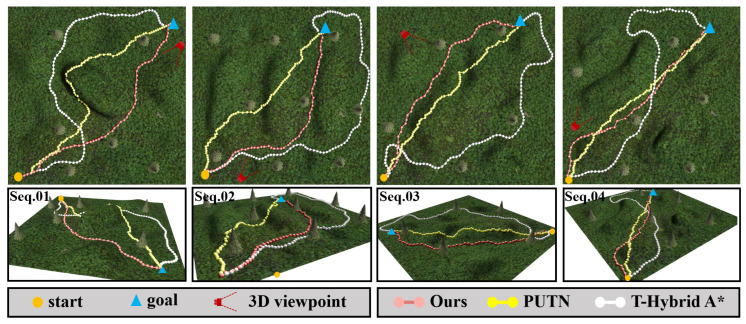
Four start-goal selected planning scenarios in simulated woodland environments for qualitative comparison of different algorithms. Each planned path is represented by a distinct color. The top row displays a top-down view of the planned paths, indicating the start (orange circle) and goal (blue triangle), along with a 3D perspective (red camera icon). The bottom row shows the 3D view from this perspective.

As shown in Figure 10, the paths generated by the PUTN algorithm mostly traversed steep slope areas and exhibited jagged paths, while T-Hybrid A* detoured around steep terrain, yielding longer trajectories. In contrast, our algorithm consistently produced safe and efficient paths across all scenarios by integrating kinematic constraints, terrain factors, path smoothness, rollover risk, and path length.

**Figure 11:**
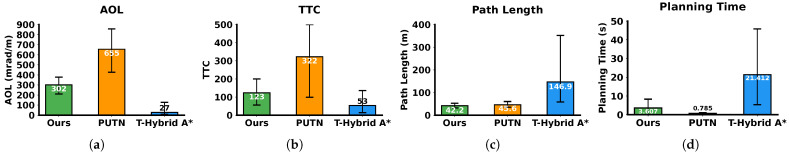
Comparison of path planning metrics using different algorithms, including (**a**) AOL, (**b**) TTC per waypoint, (**c**) total path length, and (**d**) planning time.

The quantitative results of path planning in Figure 11 corroborate these qualitative findings. The AOL of our algorithm and the average TTC of waypoints were 53.9% and 61.8% lower than PUTN, respectively. This improvement stems from the graph-search-based re-planning method within LTRs, whereas the sampling of PUTN terminated upon reaching the goal, leaving some paths unoptimized. In addition, compared with PUTN, the planned path length of our algorithm was decreased by 7.5%, reflecting both the advantages of A*-guided RRT for global path planning and the consideration of deviation cost in KT-PRM for local path planning. The T-Hybrid A* algorithm generated significant detours that increased path length by 248% and required 6× longer planning time than our algorithm. This is mainly because T-Hybrid A* evaluates its defined terrain traversability cost globally to produce minimally traversable-cost paths, but suffers from low efficiency and excessive conservativeness on maps. Overall, our algorithm efficiently produces paths with low heading variation between adjacent path segments and low TTC.

**Figure 12:**
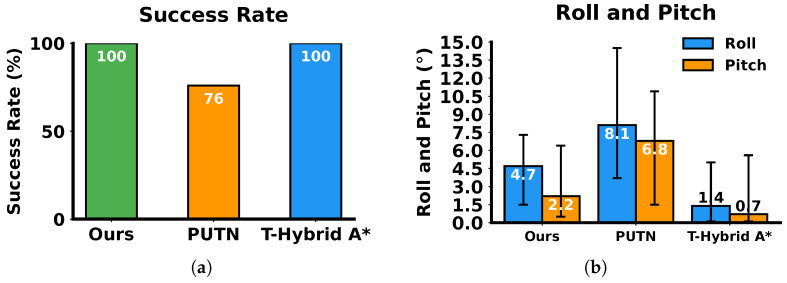
Comparison of execution metrics using different algorithms. (**a**) Success rate. (**b**) Roll angle and pitch angle of the vehicle.

After path planning with different algorithms, a simulated vehicle modeled from our FR-07 Pro vehicle (Figure 9) was executed to track the planned paths using an NMPC controller, with vehicle roll and pitch angles recorded at 0.5 s intervals. The statistical results are shown in Figure 12. Tracking paths generated by our algorithm and T-Hybrid A* both achieved 100% success, while tracking paths generated by PUTN caused several rollovers (recorded as failures) due to the high TTC of partial waypoints. Comparing with PUTN, paths generated by our algorithm reduced the average roll and pitch angles by 42.3% and 67.8%, respectively, confirming the advantages in safety of our algorithm. Similarly, T-Hybrid A* achieved relatively stable vehicle execution by planning paths that avoid high TTC regions. However, its low planning efficiency and long path lengths pose practical challenges for vehicle execution.

Overall, through both planning and tracking experiments, our three-phase algorithm, which integrates KT-PRM, demonstrated comprehensive performance through hierarchical planning. The third-phase KT-PRM comprehensively considers kinematic constraints, path smoothness, rollover risk, and path length, obtaining optimal paths via graph search on the constructed roadmap to achieve smooth, safe, and efficient paths. In path planning, our three-phase algorithm reduced AOL and terrain traversability cost compared with PUTN, and it reduced total path length and planning time compared with T-Hybrid A*. In path tracking, our algorithm achieved 100% execution success rate and low roll/pitch angles. These results confirm the excellent performance of our three-phase algorithm.

#### 4.2.2. Validity Testing of the KT-PRM Algorithm

An ablation study was conducted to verify the effectiveness of our KT-RPM algorithm for path re-planning. Based on our proposed three-phase algorithm, four variants were constructed, as summarized in Table 6. AR removes Phase 3 (KT-PRM) to evaluate its primary impact on terrain-aware safety of the planned path. ARPU substitutes Phase 3 with PF-RRT* from PUTN, which employs a sampling-based, incremental tree construction strategy without kinematic constraints. ARPRT replaces Phase 3 with PRM-T, which uses explicit point sampling and connection but considers only TTC during graph search. ARPRC extends PRM-T by augmenting the graph search cost function with motion, rollover, and deviation costs to validate the impact of comprehensive multi-factor optimization on path smoothness, safety, and length. Our algorithm employs the proposed KT-PRM, which utilizes kinematically feasible edge-pair sampling and connection in explicit graph construction, and incorporates comprehensive cost terms compared with these variants.

For each of the ten simulated maps, ten path planning experiments were conducted with the start and goal points strategically positioned at map edges. A total of 100 comparative path planning experiments were conducted between our algorithm and the four variants. Statistical analysis of corresponding metrics for both path planning and execution was conducted.

**Figure 13:**
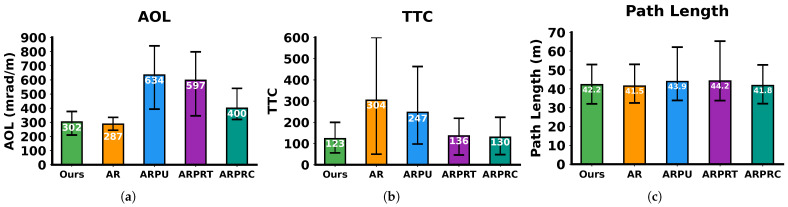
Comparison of planning path metrics under KT-PRM algorithm validity testing. (**a**) AOL. (**b**) TTC per waypoint. (**c**) Total path length.

The experimental results of path planning are shown in Figure 13. The AR algorithm, which considers only kinematic constraints while neglecting terrain factors, achieved the lowest AOL but the highest TTC. ARPU reduced TTC by 18.8% compared to AR through terrain cost integration, yet still exhibited 109.9% and 100.8% higher AOL and TTC values than our algorithm, respectively, due to its lack of kinematic constraints and the additional iterations required for optimization during incremental tree construction. ARPRT achieved a 44.9% lower TTC than ARPU, demonstrating that explicit graph construction with integrated TTC achieves exact optimality, whereas ARPU’s incremental tree construction provides only asymptotic optimization. ARPRC achieved 33% and 4.4% lower AOL and TTC values than ARPRT, respectively, through comprehensive cost terms incorporation, where the motion cost specifically yields smooth and easily traversable paths. With KT-PRM as Phase 3, our algorithm attained the lowest TTC and a sub-optimal AOL, which is only 5.2% higher than the best, but notably 24.5% lower than ARPRC. This superiority stems from KT-PRM’s consideration of deviation cost and edge-pair sampling/connection, which is fully kinematically feasible and produces smaller heading variation throughout the path compared to point sampling/connection methods. All four algorithms achieved path lengths shorter than PUTN and T-Hybrid A*, attributed to the A*-guided RRT method and LTR-confined re-planning strategy.

**Figure 14:**
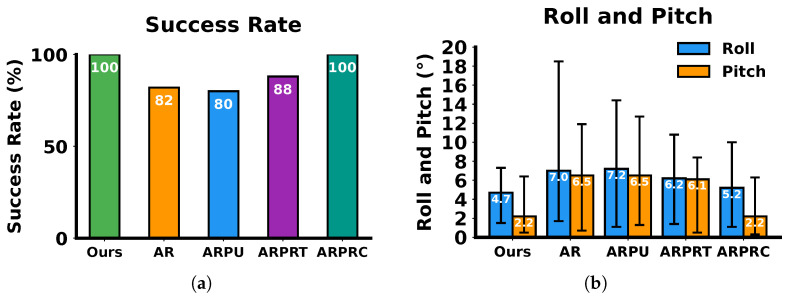
Comparison of execution metrics under KT-PRM algorithm validity testing. (**a**) Success rate. (**b**) Roll angle and pitch angle of the vehicle.

After path planning with these algorithms, path tracking experiments were conducted subsequently using the simulated vehicle with an NMPC controller. The statistical results are presented in Figure 14. The AR algorithm exhibited high rollover probabilities during tracking due to TTC neglect. ARPU and ARPRT, which lack kinematic constraints and optimize solely for TTC, still experienced rollover with larger roll and pitch angles during tracking, even when the planned paths of ARPRT exhibited low TTC values. Both ARPRC and our algorithm achieved 100% tracking success through motion cost incorporation. However, our algorithm further guarantees kinematic feasibility, robustly ensuring waypoint reachability and enhancing path smoothness compared with ARPRC.

Overall, compared with these four variants (AR, ARPU, ARPRT, and ARPRC) through both planning and tracking experiments, the KT-PRM algorithm achieves comprehensive functionality by planning with integrated consideration of kinematic constraints, TTC, motion cost, rollover cost, and path length, verifying the validity of the KT-PRM algorithm. The KT-PRM algorithm ensures kinematic feasibility through its innovative edge-pair sampling and connection method, guaranteeing reachability to planned waypoints and smoothness of the path. It integrates multiple cost terms to achieve comprehensive path optimization: TTC to steer clear of low-traversability areas, motion cost to further enhance path smoothness, and deviation cost to maintain proximity to the sub-optimal path generated from the A*-guided RRT. Finally, the graph-search-based method of KT-PRM guarantees acquisition of the exact optimal solution with respect to all cost terms. Ablation experiments validated these findings. Compared with other variants, KT-PRM demonstrates superior performance, optimizing terrain-aware metrics (TTC, success rate, and roll/pitch angles) and path smoothness (AOL).

### 4.3. Real-World Experiments

**Figure 15:**
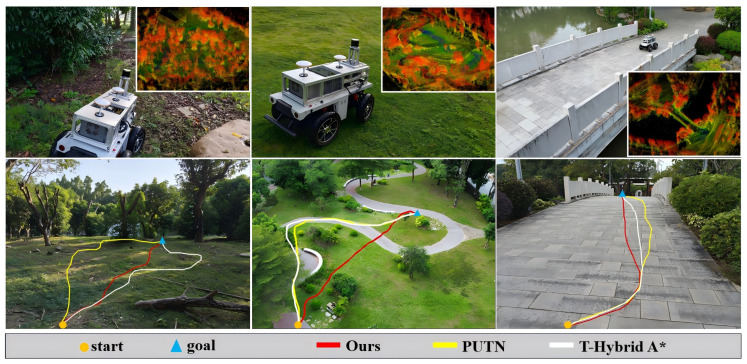
Real-world environments and three start-goal selected trajectories with three methods. The top row displays three experimental environments and constructed 3D point cloud maps (from left to right: woodland, grassland, and arch bridge). The bottom row shows the example vehicle trajectories in the corresponding environments. Each trajectory is represented by a distinct color.

We benchmarked our navigation framework against the PUTN framework and the T-Hybrid A* framework in woodland, uneven grassland, and arch bridge environments in the real world. All paths were tracked using our vehicle with an identical NMPC controller, with ten trials per environment. The experimental outcomes are presented as follows:

As shown in Figure 15, our framework and the T-Hybrid A* framework consistently achieved safe navigation across all trials. Conversely, the PUTN framework experienced two failures in the woodland environment due to wheel lift-off caused by traversal on a steep slope.

The quantitative results are presented in Table 7. Our navigation framework achieved the optimal total path length across different environments, demonstrating the inherent advantages of the A*-guided planning method. It also exhibited stable and safe execution in real environments without vehicle tilting, wheel suspension, or rollover incidents. Furthermore, our framework attained the lowest average pitch angle in the woodland environment and the lowest average roll angle in the arch bridge environment among all tested algorithms.

## 5. Conclusions

This paper proposes a navigation framework for uneven terrain. It first integrates terrain slope, flatness, elevation variation, and sparsity to generate a GTTC map. Subsequently, a three-phase path planning algorithm integrates A*, guided RRT, and the KT-PRM local re-planning algorithm, which jointly considers ground vehicle kinematic constraints, terrain factors, path smoothness, and rollover risk. This three-phase combination delivers safe and smooth global short paths over uneven terrain within relatively short planning time.

Experiments were conducted in both simulated and real-world uneven terrain environments. Through comparison with the state-of-the-art path planning algorithms on simulated uneven terrain, our three-phase algorithm integrating KT-PRM demonstrated comprehensive performance in generating safe and smooth short paths via hierarchical planning. Ablation studies further validated that KT-PRM achieves comprehensive path planning functionality compared to other variants, as its innovative edge-pair sampling and connection in roadmap construction guarantee kinematic feasibility while incorporating TTC, motion cost, rollover cost, and deviation cost into the extended accumulated cost function to obtain the optimal total cost on the roadmap. Finally, in real-world environments, compared with the PUTN framework and the T-Hybrid A* framework, our navigation framework achieved 100% safe navigation with the shortest path lengths across all scenarios, confirming its safety and efficiency. Our future work will focus on optimizing the algorithm for large-scale outdoor unstructured environments featuring dense small obstacles and complex topography. Additionally, we will develop a terrain–tyre coupled dynamic rollover model to capture the full interaction between off-road surfaces and vehicle compliance, enabling a more accurate analysis of rollover limits under real-world conditions.

## Figures and Tables

**Table 1:** Grid sampling settings.

Parameters	Range	Step
ρ	[0.5,1.9] (m)	0.01(m)
Δθ	[−1,1] (rad)	0.001(rad)

**Table 2:** Comparison of runtime across different motion cost calculation methods.

Methods	Mean Time	Min	Max
IPOPT-based	3.026 (ms)	2.737 (ms)	5.172 (ms)
Network-based	0.07 (ms)	0.057 (ms)	1.386 (ms)

**Table 3:** Hyper-parameters for sampling in A*-guided RRT.

Parameters	Value
$\rho_{s},min$	0.5m
$\rho_{s},max$	5m
$\Delta \alpha_{s},min$	$-\frac{\pi }{3}\;\mathrm{rad}$
$\Delta \alpha_{s},max$	$\frac{\pi }{3}\;\mathrm{rad}$

**Table 4:** Simulation experiment parameters.

Parameter	Value	Parameter	Value	Parameter	Value
$L_{d}$	1.2	guided RRT max iterations	5000	$\omega_{t}$	0.5
LTR threshold	0.5	KT-PRM sampling number	$N_{\mathrm{localgrid}}\;\left(\mathrm{variable}\right)$	$\omega_{m}$	0.2
$\alpha_{1}$	0.03	$\omega_{\rho }$	0.4	$\omega_{r}$	1
$\alpha_{2}$	2100	$\omega_{\theta }$	0.4	$\omega_{d}$	0.3
$\alpha_{3}$	0.01	$\omega_{c}$	0.2	vehicle max speed	1.9 m/s
$\alpha_{4}$	0.2	$\omega_{mo}$	0.5	vehicle min speed	0.5 m/s
$r_{max}$	0.1	$\omega_{a}$	0.15	NMPC prediction horizon	10
$r_{min}$	0.02	$\omega_{\kappa }$	0.6	terrain slope range	0–90°

**Table 5:** Real-world experiment parameters.

Parameter	Value	Parameter	Value	Parameter	Value
$L_{d}$	1.2	guided RRT max iterations	5000	$\omega_{t}$	0.5
LTR threshold	0.2	KT-PRM sampling number	$N_{\mathrm{localgrid}}\;\left(\mathrm{variable}\right)$	$\omega_{m}$	0.2
$\alpha_{1}$	0.03	$\omega_{\rho }$	0.4	$\omega_{r}$	1
$\alpha_{2}$	2600	$\omega_{\theta }$	0.4	$\omega_{d}$	0.3
$\alpha_{3}$	0.01	$\omega_{c}$	0.2	vehicle max speed	1.9 m/s
$\alpha_{4}$	0.2	$\omega_{mo}$	0.5	vehicle min speed	0.5 m/s
$r_{max}$	0.1	$\omega_{a}$	0.15	NMPC prediction horizon	10
$r_{min}$	0.02	$\omega_{\kappa }$	0.6	terrain slope range	0–45°

**Table 6:** Algorithm variant configurations.

Algorithm Variant	Phase 1 & 2	Phase 3			
		Name	Graph Construction Method	Kinematic Constraints	Cost Function Components
AR	✔	X	X	X	X
ARPU	✔	PF-RRT *	Incremental tree (implicit)	X	TTC
ARPRT	✔	PRM-T	points sampling and connection (explicit)	X	TTC
ARPRC	✔	PRM-C	points sampling and connection (explicit)	X	TTC, motion cost, rollover cost and deviation cost
Our Algorithm	✔	KT-PRM	edge-pair sampling and connection (explicit)	✔	TTC, motion cost, rollover cost and deviation cost

**Table 7:** Comparison of average roll angle (°), average pitch angle (°), and path length (m) in the real-world environment using different navigation frameworks. The best results are in bold.

Scenes and Metrics	Woodland			Grassland			Arch Bridge		
	Roll	Pitch	Path Length	Roll	Pitch	Path Length	Roll	Pitch	Path Length
Ours	3.68	**3.43**	**18.7**	1.33	1.36	**38.9**	**1.18**	3.86	**41.6**
PUTN	3.51	4.13	19.8	1.25	1.53	42.3	1.19	3.67	42.9
T-Hybrid A*	**3.43**	4.10	25.6	**1.09**	**1.12**	47.9	**1.18**	**3.66**	43.4

## Data Availability

Data are contained within the article.
